# Anti-*Candida* Targets and Cytotoxicity of Casuarinin Isolated from *Plinia cauliflora* Leaves in a Bioactivity-Guided Study

**DOI:** 10.3390/molecules18078095

**Published:** 2013-07-09

**Authors:** Tatiana M. Souza-Moreira, Juliana A. Severi, Keunsook Lee, Kanya Preechasuth, Emerson Santos, Neil A. R. Gow, Carol A. Munro, Wagner Vilegas, Rosemeire C. L. R. Pietro

**Affiliations:** 1 Department of Drugs and Medicines, School of Pharmaceutical Sciences, UNESP-Univ Estadual Paulista, Rodovia Araraquara-Jau, km 1, Araraquara, 14801-902, São Paulo, Brazil; E-Mails: souzatm@gmail.com (T.M.S.M.); juseveri@yahoo.com.br (J.A.S.); emersonsan@gmail.com (E.S.); 2 Department of Microbiology, Institute of Medical Sciences, University of Aberdeen, Foresterhill, Aberdeen, AB25 2ZD, Scotland, UK; E-Mails: k.k.lee@abdn.ac.uk (K.L.); kanyamt@hotmail.com (K.P.); n.gow@abdn.ac.uk (N.A.R.G.); c.a.munro@abdn.ac.uk (C.A.M.); 3 Department of Organic Chemistry, Institute of Chemistry, UNESP-Univ Estadual Paulista, R. Francisco Degni s/n, Araraquara, 14800-900, São Paulo, Brazil; E-Mail: vilegasw@gmail.com (W.V.)

**Keywords:** *Plinia cauliflora* (Myrtaceae), ellagitannin, *Candida*, antifungal activity, cell wall

## Abstract

In addition to the bio-guided investigation of the antifungal activity of *Plinia cauliflora* leaves against different *Candida* species, the major aim of the present study was the search for targets on the fungal cell. The most active antifungal fraction was purified by chromatography and characterized by NMR and mass spectrometry. The antifungal activity was evaluated against five *Candida* strains according to referenced guidelines. Cytotoxicity against fibroblast cells was determined. The likely targets of *Candida albicans* cells were assessed through interactions with ergosterol and cell wall composition, porosity and architecture. The chemical major component within the most active antifungal fraction of *P. cauliflora* leaves identified was the hydrolysable tannin casuarinin. The cytotoxic concentration was higher than the antifungal one. The first indication of plant target on cellular integrity was suggested by the antifungal activity ameliorated when using an osmotic support. The most important target for the tannin fraction studied was suggested by ultrastructural analysis of yeast cell walls revealing a denser mannan outer layer and wall porosity reduced. It is possible to imply that *P. cauliflora* targeted the *C. albicans* cell wall inducing some changes in the architecture, notably the outer glycoprotein layer, affecting the cell wall porosity without alteration of the polysaccharide or protein level.

## 1. Introduction

*Plinia cauliflora* is a tree from the Myrtaceae family [1] widespread in Brazil, Argentina and Paraguay. *P.* *cauliflora* produces an edible tasty fruit. The bark is astringent and used to treat diarrhoea and skin irritations [2]. A survey in the Brazilian Northeast area showed the popular use of its leaves and stems for the treatment of diarrhea and dysentery, while the syrup is useful to treat coughs and bronchitis [3]. Despite its importance and wide availability in Brazil, phytochemical studies on this plant are still scarce and have so far focused on the fruit [4]. Histochemical study of *P*. *cauliflora* leaves showed the presence of phenols and tannins in the midvein and smaller veins. Phenols were also verified in the palisade parenchyma and lipids in the secretory cavities. A preliminary phytochemical study of the ethanolic extract of the leaves confirmed the presence of hydrolysable tannins and flavonoids. Biological assays detected antioxidant (94.8% of radical scavenging capacity of 2,2-diphenyl-1-picryl-hydrazyl—DPPH) and antimicrobial activities, especially antifungal activity of the 70% ethanolic extract of the leaves against *Candida albicans*, *Candida parapsilosis* and *Candida* *tropicalis* (MIC of 625 µg/mL) [5]. It is well-known that *Candida* species under predisposing conditions can cause a range of infections from oral thrush and vaginitis, affecting the superficial mucosa, to candidaemia and systemic candidiasis in patients with compromised immunity [6]. The number of cases of invasive fungal infections is increasing and *Candida* species are the most common causative agents. Over 17 species of *Candida* have been reported to be etiologic agents of invasive candidiasis in humans but *C.* *albicans*, *C. glabrata*, *C. parapsilosis*, *C. tropicalis*, and *C. krusei* account for more than 90% of invasive infections [7]. In this sense, there is a great need for new antifungals with novel targets as alternative treatments to reduce the high attributable mortality rate associated with candidiasis and candidaemia and to reduce toxicity and emergence of resistance [8,9,10]. A number of plants have antifungal properties [8,10,11]. Some interesting examples include the leaves of *Phyllanthus piscatorum* and the bark of *Stryphnodendron adstringens*. The leaves are used by Yanomamï women with vaginitis and a bioactive-guided fractionation of the dichloromethane extract of *P. piscatorum* leaves led to the isolation of a lignin with a minimum inhibitory concentration (MIC) against *C. albicans* of 4 µg/mL [12]. The second example comes from a Brazilian tree (*S. adstringens*) that has bark with a high level of tannins. A polar extract was fractionated resulting in a condensed tannin enriched subfraction, which had a minimum inhibitory concentration against *C.* *albicans* of 7.8 µg/mL. The authors also demonstrated inhibition of germ tube formation, an increase in the number of buds and changes in the cell wall ultrastructure of *C. albicans* treated with that subfraction [13].

Therefore, the objective of this study was to analyze the potential of the compounds from *P.* *cauliflora* leaf extract by performing a bioassay-guided chemical study and to investigate the antifungal effect on *C. albicans* cell, as well as cytotoxic properties against a mammal cell.

## 2. Results and Discussion

**Figure 1:**
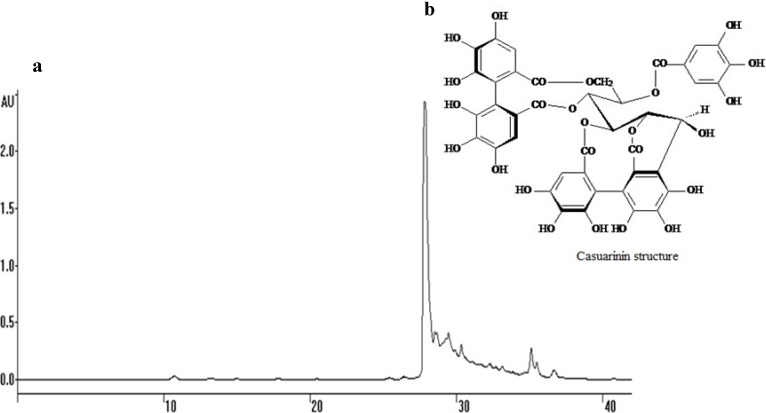
(**a**) Semi-preparative HPLC-UV chromatogram of F2 from *P. cauliflora* leaves. Chromatographic conditions: HPLC with UV detector set at 254 nm, the column was RP-18, elution phase acidified with trifluoroacetic acid 0.05% in linear gradient elution of CH_3_OH/H_2_O (20:80 in 15 min, 20:80→50:50 in 35 min, 50:50→95:5 in 40 min); flow-rate of 2.5 mL/min. (**b**) Structure of casuarinin. This hydrolysable tannin is a *C*-glycosidic ellagitannin with an open-chain glucose core called casuarinin.

The bio-guided chemical investigation of the hydroalcoholic extract from *P. cauliflora* leaves was initially carried out by fractionation of the crude extract by liquid-liquid extraction into different polarity fractions. The *n*-butanol fraction (BF) was more biologically active (see below) against *Candida* strains and fractionated further, yielding the active subfraction F2. Purification of F2 was achieved by a combination of High Speed Counter-Current Chromatography (HSCCC) and semi-preparative reverse phase High Performance Liquid Chromatography (HPLC) and led to the purification of a major hydrolysable tannin named casuarinin (Figure 1). The structure of the isolated compound was unequivocally assigned based on the ultraviolet (UV) spectral, 1D/2D nuclear magnetic resonance (NMR) and mass (MS) data, in comparison with the literature reference [14]. Casuarinin is the main ellagitannin isolated from *Casuarina stricta* [14] and was also isolated from the leaves of *Melaleuca squarosa*, a member of Myrtaceae family [15].

### 2.1. Susceptibility Testing

The in vitro susceptibility tests of the crude extract, fractions and subfractions from the leaves of *P. cauliflora* against five *Candida* strains are shown in Table 1. *C. krusei* was more susceptible to the plant samples than the other species. BF was the most active fraction, with an inhibitory effect on fungal growth with MICs in the range of 19 to 156 µg/mL, depending upon the species. This result guided us to purify and further characterize that fraction. Among the subfractions obtained by HSCCC, just one of them called F2, showed good activity against *C. albicans*, according to previous reports [16,17]. The major compound isolated from F2 by HPLC, casuarinin, had some antifungal activity and *C. krusei* was the most susceptible strain. Although casuarinin has some antifungal activity, it did not have as good activity as F2, suggesting that the minor compounds present in F2, as shown in Figure 1, also contributed to the total antifungal activity of F2. All the plant samples showed low fungicidal activity. 

Cytotoxicity was assessed indirectly using a mammalian cell line by measuring the fluorescent emission of the molecule of resazurin reduced to resorufin in response to metabolic activity. CC_50_ indicates the concentration of the plant samples that inhibited 50% of cell viability [18]. *Plinia* crude extract (PcE), BF, AF and subfraction F2 had low cytotoxic properties with a CC_50_ concentration higher than the antifungal MIC (Table 1). Casuarinin was not cytotoxic to mammalian cells at the highest concentration tested (116 µg/mL), but much higher concentrations of casuarinin were required to inhibit the fungal cells with the exception of *C. krusei* and *C. tropicalis*, which is a favorable point to study this compound for further application with animals or humans.

**Table 1:** Antifungal activity and cytotoxicity of *P. cauliflora* leaf samples, in µg/mL.

	*C.a.* (ATCC)		*C.a.* (SC5314)		*C.k.*		*C.p.*		*C.t.*		
Samples	MIC	MFC	MIC	MFC	MIC	MFC	MIC	MFC	MIC	MFC	CC_50_
Extract	156	625	156	156	19	39	78	156	312	1250	417
EAF	625	1250	NT	NT	19	78	312	625	312	625	221
BF	78	1250	156	156	19	39	19	19	156	1250	767
AF	312	>1250	NT	NT	39	78	39	78	312	1250	1500
F2	78	312	156	312	625	>1250	156	1250	312	>1250	>400
casuarinin	580	580	580	>580	26	580	580	580	145	580	>116
FCZ	4	8	2	32	32	32	4	4	32	32	NA
Amph B	0.25	0.25	1	8	0.50	0.50	0.50	0.50	0.50	2	NA

MIC and MFC results are expressed as the mode in µg/mL, after 24 h and 48 h of incubation, respectively. C.a. = *C. albicans*; C.k. = *C. krusei*; C.p. = *C. parapsilosis*; C.t. = *C. tropicalis*. EAF = ethyl acetate fraction; BF = *n*-butanolic fraction; AF = aqueous fraction; F2 = fraction 2 from BF; NT = not tested; NA = not applicable. FCZ = fluconazole (64 to 0.06 µg/mL). Amph B = amphotericin B (16 to 0.03 µg/mL). MIC test range concentrations: 1,250 to 2 µg/mL of extract and fractions and 580 to 1 µg/mL of casuarinin. CC_50_ test range concentrations: 2,000 to 4 µg/mL of extract or fractions and 116 to 0.2 µg/mL of casuarinin. CC_50_ = 50% cytotoxic concentration. Results are expressed according to the regression calculation in µg/mL.

### 2.2. Ergosterol Levels

In order to examine whether the *P. cauliflora* active fraction targets the membrane and specifically affects ergosterol levels, the ergosterol content of isolates treated with BF was measured. The sterol quantification methodology is based on the unique spectral absorption pattern produced by ergosterol at 281.5 nm and the spectral absorption pattern produced by 24(28) dihydroergosterol at 281.5 and 230 nm wavelengths. Consequently, the amount of ergosterol can be determined by calculating the total ergosterol and 24(28) dihydroergosterol content [19]. Fluconazole, which inhibits ergosterol synthesis [20] was used as a control and as expected the ergosterol content of fluconazole-treated cells was significantly decreased (Table 2). No evidence was found to implicate *P. cauliflora* acting on fungal ergosterol biosynthesis.

**Table 2:** Ergosterol quantification, in percentage (%).

SAMPLES	*C. albicans*	*C. krusei*	*C. parapsilosis*	*C. tropicalis*
Control	1.38 ± 0.32	1.13 ± 0.70	0.34 ± 0.30	1,94 ± 0.10
BF	2.03 ± 0.77(+48)	1.44 ± 0.58(+28)	0.77 ± 0.70(+29)	1,30 ± 0.86(−33)
FCZ	0.40 ± 0.23(−71) *	0.68 ± 0.08(−40)	0.05 ± 0.05(−84) *	0(−100) *

Ergosterol content expressed as a percentage (%) of the wet weight of the cell ± standard deviation. In parentheses there is the increased (+) or decreased (−) percentage value relative to the ergosterol content in control. BF = *n-*butanolic fraction. FCZ = fluconazole. * Statistically significant.

### 2.3. Reduction of P. Cauliflora Leaf Samples Antifungal Properties

#### 2.3.1. Addition of Exogenous Ergosterol

When a compound binds ergosterol, addition of exogenous ergosterol can reduce the antifungal efficacy of the compound. As a consequence, it is necessary to use a higher concentration of the compound to inhibit yeast growth relative to the MIC without exogenous ergosterol addition [8]. The effect of addition of ergosterol at 200 µg/mL to the drug susceptibility assays was determined. Deoxycholate amphotericin B can be used as positive control in this experiment since it is known to bind ergosterol in the fungal membrane and its antifungal activity is considerably compromised by the addition of 200 µg/mL exogenous ergosterol[20,21]. This finding was re-confirmed and similar results were observed with the crude extract and fractions of the plant in the present study. In our experiments, addition of exogenous ergosterol increased amphotericin B MIC 8 fold against *C. albicans* and *C. tropicalis* and 32 fold against *C. krusei* and *C. parapsilosis* (Table 3). The MIC of the extract, BF and F2 against *C. albicans* and *C. parapsilosis* increased 16 fold in the presence of ergosterol (Table 3), indicating their binding to ergosterol. 

#### 2.3.2. Sorbitol Protection Assay

Cell wall weakness can be alleviated by the addition of osmotic support, such as sorbitol [8]. In the presence of 0.8 M sorbitol, the MIC values of PcE, BF and F2 against *C. albicans* and *C. parapsilosis* were 16 fold and 4 fold higher, suggesting the plant samples may act on the cell wall. However, the susceptibility of *C. krusei* and *C. tropicalis* was not altered in presence of sorbitol. The MIC of fluconazole and amphotericin B were just 2-fold higher with sorbitol addition in accord with their target being the membrane rather than the wall (Table 3).

**Table 3:** Antifungal activity of the plant preparations in the presence of ergosterol or sorbitol, in µg/mL.

	Ergosterol				Sorbitol			
Samples	*C.a.*	*C.k.*	*C.p.*	*C.t.*	*C.a.*	*C.k.*	*C.p.*	*C.t.*
Extract	5000	39	312	625	2500	39	2500	156
EAF	5000	39	1250	625	5000	39	2500	312
BF	5000	39	1250	312	5000	39	1250	312
AF	5000	78	2500	625	5000	78	2500	1250
F2	1250	78	1250	312	1250	78	625	312
FCZ	NA	NA	NA	NA	8	64	4	64
Amph B	2	16	16	4	0.25	1	1	1

MIC results are expressed as the mode in µg/mL, for 24–48 h. C.a. = *C. albicans*; C.k. = *C. krusei*; C.p. = *C. parapsilosis*; C.t. = *C. tropicalis*. FCZ = fluconazole (64 to 0.06 µg/mL). Amph B = amphotericin B (16 to 0.03 µg/mL).Test concentrations: 5,000 to 8 µg/mL of extract. EAF = ethyl acetate fraction; BF = *n*-butanolic fraction; AF = aqueous fraction; F2 = fraction 2 from BF; NA = not applicable.

### 2.4. Ultrastructural Analysis

**Figure 2:**
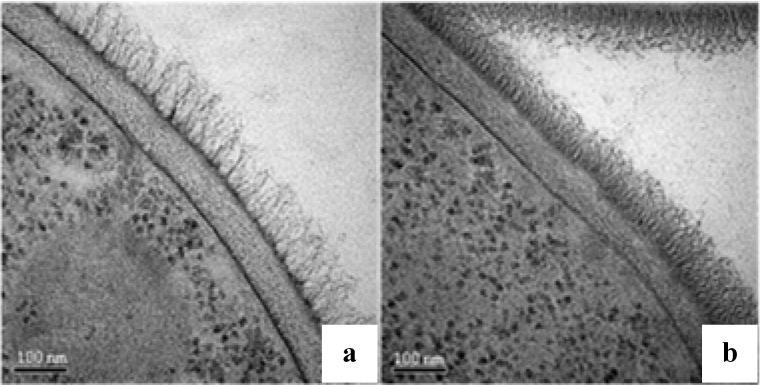
Ultrastructural analysis of the *C. albicans* yeast cell surface. (**a**) Transmission electron microscopy of cells without treatment (**b**) And cells treated with 156 µg/mL of subfraction F2 for 16 h. Magnification of 130,000×.

We also investigated whether the plant-derived compounds altered the cell wall architecture. *C. albicans* SC5314 cells were treated with subfraction F2 (156 µg/mL) and processed for transmission electron microscopy (TEM), preserving the ultrastructure of the cell wall. Untreated cells of *C. albicans* SC5314 had a typical fibrillar outer mannan layer [Figure 2(a)], but cells treated with the F2 had a denser and shorter outer layer [Figure 2(b)] indicating that the plant compounds altered cell wall structure. 

### 2.5. Assessing *C. albicans* cell wall treated with F2

To assess whether F2 did indeed target the cell wall a more detailed analysis was performed by biochemically determining cell wall composition and porosity and examining the distribution of chitin in the wall by staining with the fluorescent dye Calcofluor White (CFW). Chitin localization was not affected by treatment with the casuarinin fraction F2 (not shown). No significant changes were observed in the cell wall composition of untreated cells and cells treated with 156 µg/mL of F2 for 16 h as determined by HPAE-PAD (Table 4). As the outer fibrillar layer is rich in glycoproteins the total protein content of isolated cell walls was also measured. F2-treated yeast cell walls had a non-significant decrease in the levels of protein compared to the untreated control (Table 4). In contrast F2-treated hyphal cell walls had a significant increase (10%) in protein levels compared to the untreated samples.

**Table 4:** Cell wall assay results comparing non-treated and casuarinin fraction treated *C. albicans* SC5314.

	Control cells		Treated cells	
	Yeast	Hyphae	Yeast	Hyphae
Chitin (%)	5.1 ± 1.8	21.8 ± 1.3	4.2 ± 1.4	22.1 ± 4.0
Glucan (%)	66.3 ± 4.9	68.8 ± 0.6	73.4 ± 4.0	67.3 ± 2.0
Mannan (%)	28.6 ± 5.6	9.4 ± 1.6	22.4 ± 5.3	10.6 ± 2.1
Covalently attached cell wall protein (µg/mL)	109.4 ± 5.5	90.2 ± 5.0	114.5 ± 4.2	101.3 ± 2.0 *
Non-covalently attached cell wall protein (µg/mL)	226.5 ± 1.5	156.6 ± 0.5	227.6 ± 3.4	173.3 ± 0.1

Results of saccharide content are given as percentage (%) of total dried cell wall, while protein content is given as µg/mL and are the average ± standard deviation of triplicate samples.* Statistically significant.

The cell wall acts as a physical barrier and filter to the external environment. Changes in the cell wall architecture and cross-linking of cell wall polysaccharides to each other is likely to affect the porosity of the wall. The porosity of the cell walls of *C. albicans* treated with subfraction F2 was compared to untreated cells. Cells were treated with the polycations DEAE-dextran (500 kDa) and poly-L-lysine (50 kDa) which interact with the cell membrane releasing 260 nm UV absorbing compounds [22]. The wild type cell wall is a barrier against the free diffusion of molecules through its depth. Its porosity is a measure of its relative permeability of molecules and is normally reflective of the molecular size and charge of the permeant. Control, untreated *C. albicans* SC5314, had a relative porosity of 34.8 ± 0.2%. In comparison cells treated with the casuarinin-containing subfraction F2 had significantly reduced porosity (−9.2 ± 5.4%). The F2-treated cell wall were still as porous to poly-L-lysine but were less porous to DEAE-dextran than control cells without treatment. 

## 3. Experimental

### 3.1. General Procedures

Chromatographic equipments used were a High Speed Counter-Current Chromatography (HSCCC; P.C. Inc. Potomac, MD, USA) with a Waters 4000 constant-flow pump, a P.C. Inc. Injection Module and a Redifrac fraction collector (Pharmacia, Uppsala, Sweden) and a Varian ProStar HPLC system equipped with a RP-18 column (Phenomenex Luna). Thin layer chromatography (TLC) analyses were performed on silica gel plates (Al sheets, F_254_, 200 μm thickness, Sorbent Technologies, Norcross, GA, USA) and visualized after spraying with anisaldehyde/H_2_SO_4_ solution under UV/Vis light. NMR analyses and 2D experiments were carried out on a Varian INOVA 500 spectrometer, operating at 500 MHz for ^1^H and 150 MHz for ^13^C and chemical shifts were given in δ (ppm) using trimethylsilane (TMS) as internal standard. Complementary structural information was achieved by mass spectrometry (MS) analysis using a Thermo Finnigan TSQ Quantum Ultra AM system equipped with a hot electrospray ionization (ESI) source (electrospray voltage 3.0 kV, sheath gas: nitrogen; vaporizer temperature: 50 °C; capillary temperature: 250 °C), operating in positive and negative ionization modes. Reagents were mainly acquired from Sigma-Aldrich (St. Louis, MO, USA). Culture medium reagents were from Acumedia (Lansing, MI, USA), Sigma-Aldrich and Invitrogen (Carlsbad, CA, USA). Microbiological analysis used a VERSAmax tunable microplate reader (Molecular Devices, Sunnyvale, CA, USA), a microplate spectrofluorometer (Tecan, Männedorf, Switzerland) and a spectrophotometer (Shimadzu 1603, Kyoto, Japan). Microscopy visualization utilized a Philips CM10 transmission microscope (FEI UK Ltd, Cambridge, UK) with a Gatan Bioscan 792 (Gatan, UK, Abingdon, UK) for recording the images. Cell wall composition was analyzed by High-Performance Anion Exchange Chromatography from Dionex (Sunnyvale, CA, USA) with pulsed amperometric detection.

### 3.2. Plant Material

Leaves of *P. cauliflora* were collected in São Carlos, São Paulo, Brazil in December 2006 and identified by Marcos Sobral from the Department of Natural Sciences of the “Universidade Federal de São João Del-Rei”. A voucher herbarium specimen was deposited under number ESA 96038 at the “Herbário da Escola Superior de Agricultura Luiz de Queiroz” (ESALQ), Piracicaba, São Paulo, Brazil.

### 3.3. Extraction and Isolation

The leaves were dried at 40 °C and powdered using a knife mill. The crude extract was obtained by percolation of 80 g of the powder with 70% aqueous ethanol (1L, 3 days, ambient temperature). The organic solvent was evaporated at 40 °C under reduced pressure and the aqueous residue lyophilized, yielding a green crude extract (30%). A portion (15 g) of the extract was suspended in water (500 mL) and partitioned successively (v/v, 3 × 500 mL each) with ethyl acetate, followed by *n*-butanol, yielding the ethyl acetate fraction (EAF, 21.8%), *n*-butanol fraction (BF, 41.7%) and aqueous fraction (AF, 36.5%). The BF was fractionated by preparative High Speed Counter-Current Chromatography equipped with a multilayer helical column containing two coils (φ = 1.68 mm, internal extreme β = 0.50, external extreme β = 0.85, revolution ratio n = 10 cm). An amount of 1.25 g of BF was dissolved in 15 mL of the mobile phase composed by a solution of ethyl acetate-*n*-butanol-water (3.8:1.2:5, v/v), centrifuged and the supernatant injected at a flow-rate of 1.5 mL/min. Rotation was set up at 850 rpm and a total of 130 fractions (4 mL each) were collected and analysed by TLC (CHCl_3_/MeOH/PrOH/H_2_O (5:6:1:4, v/v/v/v - organic phase, anisaldehyde/H_2_SO_4_ reagent for detection) and grouped according to their TLC profile. The active-tannin enriched subfraction F2 (100 mg) was sequentially purified by reverse-phase using a HPLC system equipped with a RP-18 column (250 × 4.6 mm i.d., 5 µm), mobile phase acidified with trifluoracetic acid (TFA) 0.05% in linear gradient elution of CH_3_OH/H_2_O as follow: 20:80 in 15 min, 20:80→50:50 in 35 min, 50:50→95:5 in 40 min; eluted at a flow-rate of 2.5 mL/min. The effluent was monitored using a ProStar 330 photodiode-array ultraviolet detection system at 254 nm and led to the isolation of the compound casuarinin (**1**, 13 mg) [14]. Colorless amorphous powder. UV λ_max_ nm: (MeOH): 213, 232, 257sh. ^1^H-NMR (DMSO-*d*_6_): *δ* 6.94 (2H, d, *J *= 2 Hz, galloyl ArH), 6.57, 6.29, 6.25 (1H each, s, HHDP), 5.39 (1H, dd, *J *= 5.0, 2.0 Hz, H-1), 5.26 (1H, dd, *J *= 8.0, 2.0 Hz, H-4), 5.21 (1H, dd, *J* = 2.0, 8.0, H-3), 5.17 (1H, m, H-5), 4.56 (1H, m, H-2) 4.53 (1H, m, H-6), 4.00 (1H, d, *J *= 13 Hz, H-6). ESI-MS (positive mode) *m/z* 937 [M+H]+. ESI-MS (negative mode) m/z 935 [M-H]-. C_41_H_28_O_26_. Those experiments were performed at the Institute of Chemistry and the School of Pharmaceutical Sciences, UNESP, BRA

### 3.4. Fungal Strains, Cell Line and Culture Conditions

The fungal strains used were *Candida albicans* ATCC 64548, *Candida krusei* ATCC 6258, *Candida parapsilosis* ATCC 22019 and *Candida tropicalis* ATCC 750 and one clinical isolate *Candida albicans* SC5314. Each strain was maintained on YPD agar (1% (w/v) yeast extract, 2% (w/v) mycological peptone, 2% (w/v) glucose, 1.6% (w/v) agar) and cultured in YPD broth at 30 °C with shaking at 200 rpm for 16 h to prepare inocula for experiments. When required, the strains were cultured in Sabouraud broth (1% (w/v) mycological peptone, 2% (w/v) glucose). Minimal inhibitory concentration (MIC) was performed with RPMI 1640 medium. Hyphal growth of strain SC5314 was induced using two different growth media: water with 20% fetal calf serum (FCS) and Lee’s medium [23,24] and incubation at 37 °C. The rabbit corneal cell line (ATCC SIRC CCL-60) was maintained in Eagle’s MEM containing 10% (v/v) fetal bovine serum, 2 mM L-glutamine and 0.2% sodium bicarbonate. The cultures were incubated at 37 °C with 5% CO_2_ until reaching confluence. Microbiological and cytotoxic assays were realized at School of Pharmaceutical Sciences in UNESP, BRA and experiments with *Candida albicans* SC5314 were performed at the Institute of Medical Sciences, University of Aberdeen, UK, including TEM analysis, MIC, hyphal growth and cell wall assays.

### 3.5. Antifungal Susceptibility Testing

Minimal inhibitory concentration (MIC) was determined using the broth microdilution technique according to the Clinical and Laboratory Standards Institute recommendations [25]. Solutions of *P. cauliflora* extract and fractions were prepared in DMSO and serially diluted (<1% DMSO) to obtain concentrations of 1,250 to 2 µg/mL of extract and fractions and 580 to 1 µg/mL of the pure compound. The inocula were adjusted to a density of 2.5 × 10^3^ cfu/mL in each well and the microplates were incubated in RPMI 1640 medium at 35 °C for 24 h. The endpoint of antifungal activity was interpreted as the lowest concentration resulting in an observable inhibition of growth compared to the no drug control cultures. In addition, for *C. albicans* SC5314 the endpoint reading was measured at an optical density (OD) of 405 nm in a microplate reader [26]. Fluconazole and deoxycholate amphotericin B were utilized as control drugs. The MIC of PcE, BF, F2 and amphotericin B were also determined in the presence of 0.2 µg/mL ergosterol or 0.8 M sorbitol [8]. This MIC was determined after 24–48 h of incubation at 35 °C. Minimal fungicidal concentration (MFC) was defined as the concentration at which the number of colony forming units was zero after 2 µL of microplate cultures were incubated at 37 °C for 48 h on YPD agar [26]. Data are presented as the mode values of triplicates.

### 3.6. Cytotoxicity Testing

The mammalian cytotoxicity of *P. cauliflora* samples was evaluated as follows: aliquots (300 µL) of a 1×10^5^ cells/mL SIRC cell suspension was added to the wells of 96-well microplate and incubated in Eagle’s MEM containing 10% (v/v) fetal bovine serum, 2 mM L-glutamine and 0.2% sodium bicarbonate at 37 °C, 5% CO_2_ for 72 h. The medium was removed and replaced with fresh medium containing extract or fractions (2,000 to 4 µg/mL) and casuarinin (116 to 0.2 µg/mL) and the plate incubated at 37 °C, 5% CO_2_ for 24 h. A 0.1 mg/mL resazurin solution was added and after 3 h of incubation the fluorescence of the reduced product resorufin was read using a microplate spectrofluorometer with excitation and emission wavelengths of 530 nm and 590 nm, respectively [18,27]. Then 50% cytotoxic concentration (CC_50_) was calculated [28]. The test was done in triplicate.

### 3.7. Sterol Quantification Method

Total cellular ergosterol of the standard strains treated with BF was quantified [19]. One colony was incubated overnight in Sabouraud broth and used to inoculate (2.5 × 10^3^ cfu/mL) Sabouraud containing BF at MIC value. After incubation at 35 °C, 18 h, 135 rpm, cells were collected, washed and saponified. Sterols were extracted with hexane. An aliquot of sterol extract was diluted 5-fold in 100% ethanol and scanned in a spectrophotometer between 200 and 300 nm. The ergosterol content was calculated as percentage according to the equations in Arthington-Skaggs *et al*. [19]. The test was done in triplicate and compared with the results obtained for fluconazole.

### 3.8. Ultrastructural Analysis by Transmission Electron Microscopy (TEM)

Cells were inoculated (OD600 < 0.2) into YPD broth plus 156 µg/mL subfraction F2 and incubated for 16 h at 30 °C, 200 rpm. The cells were filtered and humidified samples of untreated and treated cells were frozen by high pressure (Leica EM PACT high-pressure freezer, Vienna, Austria). Freeze substitution, sectioning and visualization were done as described previously [29].

### 3.9. Cell Wall Polymer Quantification

The cell walls were prepared from untreated (control) and treated (156 µg/mL of F2) yeast and hyphal *C. albicans* SC5314 cells grown as follows. For yeast growth, 1 mL from overnight YPD culture (OD at 600 nm < 0.2) was inoculated in 100 mL YPD and incubated for 16 h at 30 °C, 200 rpm. To induce hyphae formation cells were washed in H_2_O and inoculated at <2 × 10^7^ cells/mL into 200 mL of 20% FCS in sterile distilled water and incubated for 16 h at 37 °C, 200 rpm. Cells were harvested by centrifugation and cell walls were isolated as described [30].

Chitin, β-glucan and mannan levels were determined by quantification of glucosamine, glucose and mannose after acid hydrolysis using High-Performance Anion Exchange Chromatography with pulsed amperometric detection (HPAE-PAD). Monosaccharides were separated on a CarboPac PA-10 column and eluted isocratically with 12 mM NaOH [24]. The total μg per 1 mg of each cell wall component was obtained by calibration using standard curves of glucosamine, glucose and mannose, and expressed as a percentage of total dry cell wall. All data are presented as the mean values of triplicate experiments. The amount of protein in cell wall fractions was determined by Bradford method [30]. All data are presented as the mean values of triplicate samples.

### 3.10. Relative Cell Wall Porosity

Cells from an overnight YPD culture of *C. albicans* SC5314 were inoculated at OD600 < 0.2 into 50 mL YPD broth and incubated for 3 h at 30 °C, 200 rpm. The F2 subfraction was added (final concentration 156 µg/mL) and cells were incubated for 2 h. The cells were harvested and washed with water. Cells at a density of 1 × 10^8^ cells/mL were dispensed in duplicate in 1 mL aliquots, centrifuged and 10 mM of Tris-HCl buffer pH 7.5 was added to each of the cell pellets, which were divided in two sets of reactions: one received 1 mL of 5 µg/mL DEAE-dextran and the other 1 mL of 15 µg/mL poly-L-lysine. The samples were incubated for 30 min at 30 °C, 200 rpm, centrifuged at 16,000 × g for 3 min, the supernatant collected and again centrifuged. The supernatant absorbance was measured at 260 nm. Relative porosity was calculated as [(A_260_DEAE-dextran—A260buffer)/(A_260_poly-L-lysine—A260buffer) × 100]. Each test was performed in triplicate [22].

### 3.11. Statistical Analysis

Where necessary, statistical analysis was performed using ANOVA. A Dunnett’s T-test was used to compare treated with non-treated cells in the assays of hyphal growth, sterol and cell wall polymers quantification and cell wall porosity. A 5% significance level was adopted.

## 4. Conclusions

It can be proposed that the specific cell wall changes induced by treatment with subfraction F2 may be due to the formation of a tannin-cell wall protein complex on the outermost layer of *C. albicans* since tannins are known to complex with macromolecules such as proteins and polysaccharides [31]. To corroborate these potential effects of F2 we observed: (i) a denser outer mannoprotein layer visualized by TEM indicating precipitate formation; (ii) no changes in mannan content and iii) reduced cell wall porosity. Hence, the present study proposes that the tannin-rich fraction F2 of *P. cauliflora* has the ability to interfere with the outer glycoprotein-rich layer of *C. albicans*. 
